# Trends and Dietary Assessment According to Fruit and Vegetable Intake in Korean Elderly People: Analysis Based on the Korea National Health and Nutrition Examination Survey 1998, 2008, and 2018

**DOI:** 10.3390/foods9111712

**Published:** 2020-11-22

**Authors:** Yong-Seok Kwon, Jihye Ryu, Yuyeong Yang, Yoo-Kyoung Park, Sohye Kim

**Affiliations:** 1 National Institute of Agricultural Sciences, 166 Nongsaengmyeong-ro Wanju-gun, Jeollabuk-do 55365, Korea; selenium2012@korea.kr (Y.-S.K.); jhryu13@korea.kr (J.R.); eweew32@korea.kr (Y.Y.); 2 Department of Medical Nutrition, Graduate School of East-West Medical Science, Kyung Hee University, Yongin 17104, Korea; ypark@khu.ac.kr; 3 Nutrition Care Services, Seoul National University of Bundang Hospital, Seongnam 13620, Korea

**Keywords:** vegetable, fruit, elderly, trend, KNHANES

## Abstract

The study aimed to examine the 20-year trends in fruit and non-starch/unsalted vegetable intake among the Korean elderly aged 65 years or older based on the Korea National Health and Nutrition Examination Survey (KNHANES) data. A total of 3722 elderly citizens aged 65 years or older who participated in the dietary survey (24-h recall of dietary intake) of the 1998, 2008, and 2018 NHANES were selected as the subjects of this study. Fruit and non-starchy/unsalted vegetable intake increased by approximately 86.53 g over the past 20 years, from 268.27 g in 1998 to 355.8 g in 2018. In particular, 65–74-year-olds had an increased intake by approximately 130.38 g over the past 20 years, from 277.34 g in 1998 to 407.72 g in 2018. In addition, snacks intake significantly increased over the past 20 years (*p* for trend < 0.001). Intake according to daily meal cooking location increased by approximately 130 g over the past 20 years, from 64.50 g in 1998 to 123.39 g in 2008, and to 198.01 g in 2018. The annual proportion of the total elderly population who meet the amount of vegetable food intake recommended by the World Health Organization (WHO)/World Cancer Research Fund (WCRF) (400 g or more fruits and non-starchy vegetables) increased by approximately 11.28%p (percentage points) over the past 20 years, from 21.78% in 1998 to 24.63% in 2008, and to 33.06% in 2018. The results of this study suggest that more fundamental measures are required to increase the fruit and non-starchy vegetable intake among the elderly. Furthermore, it is thought that the results of this study can be used as basic data in establishing dietary policy. In addition, it is thought that it can be used in developing nutrition education and dietary guidelines for enhancing fruit and vegetable intake.

## 1. Introduction

With the improvement of household income, living standards and advances in medical technology, the number of elderly people is increasing significantly along with the extension of life expectancy. The trend is accelerating in the direction of a sharp increase in the proportion of elderly people around the world, and the population is aging in Korea as well. As of 2015, the number of elderly people aged 65 or older was about 6.66 million, accounting for 13.1% of the total population and 14.3% as of 2017. This percentage will continue to increase in the future, and the percentage of the population aged 65 and over is expected to more than double in 2030 and reach 40.1% in 2060 [1,2]. Therefore, there is an increasing interest in the health and dietary life of the elderly. With increase in the elderly population in Korea, elderly people’s diet is the focus area. A previous study revealed that some elderly citizens have malnutrition owing to decreased physiological and digestive functions. A study conducted on nutrient intake status in each stage of the life cycle reported that the elderly and adolescents were nutritionally vulnerable compared to other age groups [3]. Moreover, according to Korea’s 2018 National Health Statistics, the elderly’s intake of nutrients, especially fat, calcium, vitamin A, riboflavin, niacin, and vitamin C, was reported to be low [4]. Particularly, sufficient intake of fruits and vegetables, which are dietary sources of minerals and vitamin C, provides resistance to diseases and slows disease progression, as it prevents obesity by lowering blood pressure and fat absorption; lowers the risk of cardiovascular disease and cancer; provides emotional stability by promoting nervous system functions; and relieves stress [5]. The US Health Professionals’ follow-up study reported a lower risk of cardiovascular diseases with increased intake of fruits and vegetables [6], and the European Prospective Investigation into Cancer and Nutrition (EPIC) study reported that a group of diabetes patients who consumed a lot of vegetables and fruits had a lower risk of cardiovascular diseases [7]. As dietary sources of micronutrients, such as minerals and vitamin C, fruits and vegetables aim at preventing diseases in the elderly. 

Looking at national and international recommended intakes of vegetables and fruits, the National Cancer Institute in the United States recommends eating at least five servings of fruits and vegetables per day in its “5 A Day for better Health (5 A Day)” program [8], and the World Cancer Research Fund (WCRF)/American Institute for Cancer Research (AICR) recommends eating at least 400 g of vegetables and fruits per day [9]. In Korea, the nutrition section of the 4th National Health Promotion Plan (2016–2020) aims at increasing the percentage of the population consuming more than 500 g of vegetables and fruits per day to 41.2% by 2020 [10]. However, Korea’s 2018 National Health Statistics reported that the total daily intake of vegetables and fruits of the elderly aged 65 or older was 458.4 g (fruit: 169.6 g, vegetables: 288.8 g), which was less than 500 g [4]. A number of previous studies have reported that intake of vegetables and fruits is important for maintaining good health and preventing chronic diseases in adults [11,12,13,14]. However, most studies on the intake of vegetables and fruits with a focus on the elderly included only some sample subjects, and no extensive study has been conducted yet, except a study on vegetable intake or vegetable and fruit intake in all Koreans participating in the Korea National Health and Nutrition Examination Survey (KNHANES) [15,16] and a study on the vegetable and fruit intake among adults [17,18,19,20,21]. No in-depth analysis of vegetable and fruit intake among elderly Koreans has been conducted either.

Therefore, this study aimed to analyze the vegetable and fruit intake status of elderly Koreans using data from the KNHANES, which is a national database representing the dietary life in Korea. Other reasons for using the KNHANES data are that the data have been accumulated for 20 years since its inception in 1998 and that it is also important to examine how the intake of fruits and vegetables of elderly subjects has changed over time because food intake is affected by the society and economy. This study aimed to examine the trends in fruit and non-starch/unsalted vegetable intake of elderly Koreans aged 65 or older over the past 20 years using the 1998, 2008, and 2018 National Health and Nutrition Survey data. Furthermore, this study aimed to observe the factors influencing fruit and vegetable intake every 10 years to propose a plan to lead a balanced diet for the elderly and serve as the basis for nutrition education or dietary policy.

## 2. Materials and Methods 

### 2.1. Composition of Korea National Health and Nutrition Examination Survey Data

The KNHANES is a large-scale statistical survey conducted by extracting a representative sample from the entire country’s population to comprehensively understand the health and nutritional status of people, used as basic data for health policies for improving nutrition, preventing diseases, and developing health promotion programs [22]. KNHANES is conducted by extracting sampling survey zones each year. It was conducted from November to December in 1998 (Phase 1) and 2001 (Phase 2); from April to June in 2005 (Phase 3); and from July to December in 2007 (Year 1 of Phase 4). Since 2008 (Year 2 of Phase 4), the survey has been conducted throughout the year [23]. KNHANES involves a health survey, a medical examination, a nutrition survey, etc., among which, the nutrition survey aims to determine the food and nutritional intake level and eating habits of Koreans and consists of a dietary life survey, a food frequency questionnaire, a dietary intake survey by the 24-h recall method, and a food security survey [22].

### 2.2. Study Subjects

**Figure 1:**
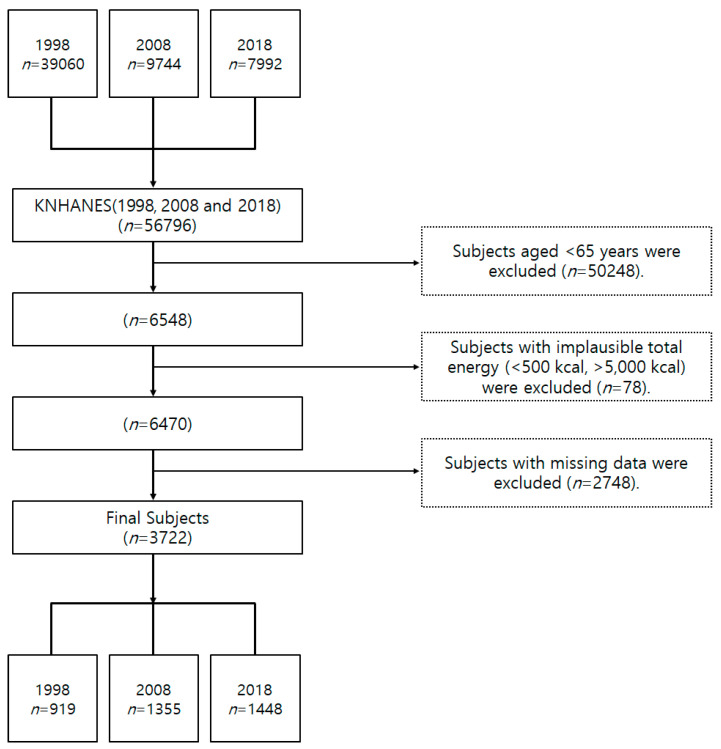
The flow chart regarding subject samples of this study.

To examine the trends in fruit and vegetable intake of the elderly over the 20 years, from 1998 to 2018, this study selected adults aged 65 years or older who participated in the dietary intake survey of KNHANES conducted by the 24-h recall method. Among them, cases with the total caloric intake per day were less than 500 kcal or more than 5000 kcal were excluded. Furthermore, subjects who did not participate in the dietary survey were excluded from this study (Figure 1). A total of 3722 subjects were selected as final subjects for the study: 919 subjects from Phase 1 (1998), 1355 subjects from Phase 2 (2008), and 1448 subjects from Phase 3 (2018). KNHANES was carried out with the approval of the Research Ethics Review Board of the Korea Centers for Disease Control and Prevention (approval number: 2008-04EXP-01-C, 2018-01-03-P-A).

### 2.3. Fruit and Vegetable Intake

Vegetable and fruit intake was classified using the food code variables and food intake variables of the 24-h recall data as based on previous studies [20,24,25]. In general, vegetable and fruit juices were excluded from the fruit and vegetable food intake since the exact intake cannot be determined due to the high water content as reported by Kwon et al. [20]. Starchy vegetables, such as potatoes and sweet potatoes, were excluded from vegetable intake according to the World Cancer Research Foundation (WCRF) and World Health Organization (WHO) vegetable food intake guidelines. After the primary classification, vegetable intake was again classified into “total vegetable intake,” “salted vegetable intake (kimchi, seasoned vegetable salads, pickles and other salted vegetables)” and “non-starchy/unsalted vegetable intake.” Fruit intake was calculated as the total fruit intake, of which “candied fruit intake” included the intake of sweetened fruit preserves and jams and “fresh fruit intake” included all other fruit intake excluding the intake of sweetened fruit preserves and jams. Finally, the “fresh fruit and non-starchy/unsalted vegetable intake” including non-starchy/unsalted vegetable intake and fresh fruit intake was named “fruit and vegetable” for comparison with the WCRF and WHO vegetable food intake guidelines [9,26]. In this study, intake of fruits and vegetables was classified into seven categories: “total vegetable intake”, “‘unsalted/non-starchy vegetable intake”, “salted vegetable intake”, “total fruit intake”, “fresh fruit intake”, “candied fruit intake”, and “fruit and vegetable intake”.

### 2.4. Covariate

Subjects’ general characteristics were analyzed such as gender, age, level of education, residential area, household income, family size and chewing ability. The basic variables included in KNHANES were used without changes, such gender, age, residential area, and level of household income. Level of education, employment status, family size, weight status, chewing ability, and age were appropriately modified for this study based on the health survey data. First, in terms of the level of education, subjects with elementary level or lower education and middle school graduates were integrated into the same category of “middle school or lower,” and the rest were classified into “high school” and “college or higher.” Residential area was classified into urban area and suburban/rural area; employment status was classified into occupation (employed) and inoccupation (unemployed), and family size was divided into single-person household and multi-person household (two or more). Second, BMI (Body Mass Index) was used to classify weight status. Based on WHO and Asia-Pacific obesity guidelines, BMI <18.5 kg/m^2^ was classified as underweight, 18.5–23.0 kg/m^2^ as normal, 23.0 kg/m^2^–25.0 kg/m^2^ as overweight, and 25.0 kg/m^2^ or heavier as obesity group [27]. Age was classified into 65–74 and 75 or older. Finally, for chewing ability, a relevant questionnaire item has been included in KNHANES since Year 1 of Phase 4 (2007), and it was classified as is in the questionnaire item: “very uncomfortable”, “uncomfortable”, “normal”, “not uncomfortable”, and “not uncomfortable at all”.

### 2.5. Dietary Behavior

Dietary behavior was analyzed in terms of daily meal, type of meal according to eating place, eating-out frequency, and food security. To analyze the intake, daily meal (variable name: n_meal) was classified into breakfast, lunch, and dinner. Subjects who selected snacks (variable value: 4) were classified as having snacks and the rest as not having snacks. Based on the eating-out status variable (variable name: n mtype) used in the study by Chung et al. [28], the type of meal according to cooking location of daily meal was classified into eating at home (home-prepared meals, home-prepared lunches, meals prepared by neighbors or relatives, etc.), eating at commercial locations (Korean restaurants, Chinese restaurants, Japanese restaurants, snack bars, bakeries/patisseries, stalls/shops, instant foods, fast foods, fresh cut products, generic food products and other food items, etc.) and using institutional food services (school meals, workplace meals, preschool/kindergarten meals, seniors’ meals, free meals, temple/religious meals, and other meals). Eating-out frequency (variable name: L_OUT_FQ) was classified by modifying the questionnaire item of the dietary life survey. “Once a day” and “twice a day or more” were integrated into “at least once a day”; “Once or twice a week”, “3–4 times a week” and “5–6 times a week” were integrated into “1–6 times a week”; and “1–3 times a month”, and “rarely (less than once a month)” were used without changes. Food security was classified based on the questionnaire item “Which of the following best describes the eating habits of the family over the past year?” which was newly introduced to KNHANES in 2005 [29,30]. The answer “enough foods of various types provided for every member of the family” was classified as enough food secure; “enough foods of not as various types provided for every member of the family” as mildly food insecure; and “insufficient foods provided due to financial difficulties” as severely food insecure.

### 2.6. Statistical Analysis

SAS (Statistical Analysis System, SAS Institute, Cary, NC, USA) ver. 9.4 was used for all analyses, and the statistical significance level set to α = 0.05. Because the KNHANES data are based on multistage stratified cluster sampling, the analysis was performed considering the strata variable (Kstrata), cluster variable (Primary Sampling Unit, PSU), and weight (the time series weight Wt_ntr_t was used for the data from 1998, and Wt_ntr_t for the rest of the years). Categorical variables, such as general characteristics of survey subjects and their dietary behavior, were expressed as frequency (*n*) and weighted percentage (Weighted %) using frequency analysis, and the significance was tested using chi-square test. Continuous variables, such as fruit and vegetable intake, were expressed as means and standard errors using descriptive analysis, and p for trend, which is the significance value of a lineal trend, was calculated using PROC SURVEYREG to test the significance. At this time, the fruit and vegetable intake by gender was corrected using age and energy intake; the fruit and vegetable intake by age was corrected using gender and energy intake; and gender, age, and energy intake were used to analyze all other fruit and vegetable intakes. Correlation analysis was conducted to analyze the relationship between vegetable and fruit intake and nutrient intake. Because the SAS program does not have a command for correlation analysis for data collected by multistage stratified cluster sampling, only the weight variable was applied for the analysis. The general characteristics of subjects were set as independent variables for the analysis of factors affecting the annual increase and decrease in population consuming over 400 g of fresh fruits and non-starch/unsalted vegetables per day, as recommended by the WHO and WCRF. The group of subjects consuming over 400 g of fruits and vegetables was classified as “1,” and the group not meeting the standard classified as “0”, and both applied as dependent variables. Multiple logistic regression analysis was used to calculate the odds ratio (OR) and the 95% confidence interval (CI). In this analysis, energy intake was used as a correction variable.

## 3. Results

### 3.1. Fruit and Vegetable Intake by Year

Table 1 shows the fruit and vegetable intake by year. The ratio of salted vegetable intake to total food intake showed a significant decrease over the years, from 13.31% in 1998 to 13.36% in 2008, and to 9.98% in 2018, with unadjusted and adjusted *p* for trend (*p* for trend < 0.001). The ratio of subjects consuming over 400 g/day of fruits and non-starch vegetables as recommended by WHO and WCRF to the total number of subjects showed a significant increase by 3.02%p (percentage points) over the years, from 23.98% in 1998 to 27.0% in 2018, with unadjusted and adjusted p for trend (*p* for trend < 0.001). The intake of these subjects significantly increased by approximately 86.53 g/day over the past 20 years, from 268.27 g/day in 1998 to 355.8 g/day in 2018, with unadjusted and adjusted p for trend (*p* for trend < 0.001).

### 3.2. General Characteristics of Survey Subjects according to Survey Year

Table 2 shows the general characteristics of survey subjects according to survey year. There was a significant difference in age (*p* < 0.001). Age 65–74 years showed a decrease by approximately 10%p (percentage points) over the past 20 years from 67.10% in 1998 to 57.40% in 2018, whereas age 75 or older increased by approximately 10%p (percentage points) over the 20 years (from 32.90% in 1998 to 42.60% in 2018). As for family size, the number of single-person households tended to increase by about 5 %p (percentage points) from 15.87% in 1998 to 20.24% in 2018, whereas the number of multi-person households consisting of two or more family members tended to decrease by about 5%p (percentage points) from 84.13% in 1998 to 79.76% in 2018 (*p* = 0.0003). The residential area showed a significant difference over the past 20 years (*p* = 0.0003). The number of subjects residing in urban areas increased by about 22.0%p (percentage points) over the 20 years, from 56.69% in 1998 to 78.66% in 2018, and the number of subjects residing in rural areas decreased by approximately 21.97%p (percentage points). As for education level, the number of subjects with high school or lower education tended to decrease from 1998 to 2018, and the number of subjects with college or higher education increased by about 10%p (percentage points) from 2.85% in 1998 to 12.19% in 2018 (*p* < 0.001). In terms of weight, the ratios of underweight and normal body types to the total number of subjects decreased over the past 20 years, while the ratios of the subjects in the overweight and obesity groups increased by approximately 6.8%p (percentage points) to 12.2%p (percentage points) (*p* < 0.001). There was no significant difference in chewing ability by year. “Very uncomfortable” decreased by about 21.27%p (percentage points), from 2008 to 2018; “uncomfortable” showed almost no difference; “normal” increased by approximately 10.0%p (percentage points); “not uncomfortable” decreased by approximately 2%p (percentage points); and “not uncomfortable at all” increased by about 10%p (percentage points).

### 3.3. Fruit and Vegetable Intake by General Characteristics According to Survey Year

Table 3 shows the fruit and vegetable intake according to the general characteristics by year. Considering gender, age (65–74 years), residential area, employment status, education level (except collage level or higher education), weight status (except underweight), “not comfortable” chewing ability, household income, and family size, there were significant increases of 31–131 g/day over the past 20 years, with unadjusted and adjusted *p* for trend (*p* for trend < 0.001). Specifically, in terms education level, only the “‘college or higher” group showed no significant increase as this group continued to show a high intake of 400 g/day or more over the past 20 years from 551.81 g/day in 1998 to 467.66 g/day in 2008 and 543.78 g/day in 2018. In terms of wright status, “normal,” “overweight,” and “obesity” showed significant increases by year, except “underweight.” In terms of chewing ability, “normal” and “not comfortable” with unadjusted p for trend showed significant increases. After adjusting p for trend by gender, age, and energy intake, only “not uncomfortable” showed a significant increase in the amount of fruit and vegetable intake. As for the household income variable, the “middle-high” and “high” groups showed no significant increases after adjusting p for trend by gender, age, and energy intake, but the “low” and “middle-low” groups showed significant increases. All groups of “single-person households” and “multi-person households” showed significant increases. The amount of fruit and vegetable intake was higher in multi-person households than in single-person households.

### 3.4. Dietary Behavior of Survey Subjects According to Survey Year

Table 4 shows the dietary behavior of survey subjects by year. There were significant differences in variables including eating breakfast, eating lunch, eating at home, eating at commercial location, and using institutional food services from 1998 to 2018 (*p* < 0.05). Looking at some of these variables, the ratio of the subjects eating no breakfast increased by about 6%p (percentage points) from 1998 and 2018. There was no significant difference in terms of the number of subjects eating snacks. It showed an increase of about 20%p (percentage points) over the past 20 years, as 40.58% of the subjects answered “Yes” in 1998, and 60.28% in 2018. The proportion of subjects eating at commercial location increased by about 45%p (percentage points) over the past 20 years, as the subjects who answered “Eaten” increased from 27.11% in 1998 to 72.52% in 2018. In terms of food insecurity, no survey was conducted in 1998, and there was a significant difference over the past 10 years from 2008 to 2018 (*p* < 0.001). The proportion of subjects who belonged to the “enough food secure” group increased by about 16%p (percentage points) over the past 10 years from 33.48% in 2008 to 49.59% in 2018. In terms of eating-out frequency, the proportion of subjects who ate out less than once a month decreased by about 47%p (percentage points) over the past 20 years, from 67.04% in 1998 and 20.39% in 2018, whereas the proportion of subjects who ate out 1–6 times a week increased by about 34%p (percentage points) over the past 20 years, from 9.51% in 1998 and 43.95% in 2018.

### 3.5. Fruit and Vegetable Intake by Dietary Behavior according to Survey Year

Table 5 shows the fruit and vegetable intake by dietary behavior according to survey year. As a result of analyzing the ratio of the intake according to daily meal to the total fruit and vegetable intake, the ratio of snacks consumption significantly increased over the past 20 years (*p* for trend < 0.001). It increased by about 8%p (percentage points) over the past 20 years from 25.57% in 1998 to 33.48% in 2018. In addition, there was a significant increase in fruit and vegetable intake in breakfast and lunch, while there was no significant increase in fruits and vegetable intake in dinner. Looking at the ratio of cooking location of daily meal to the total fruit and vegetable intake, the ratio from eating at home significantly decreased (*p* for trend < 0.001). Looking at the intake, the fruit and vegetable intake at home also significantly decreased from 198.39 g in 1998 to 157.08 g/day in 2008, and 150.32 g/day in 2018. In contrast, the ratio from commercial locations increased by about 30%p (percentage points) over the past 20 years (*p* for trend < 0.001). The intake increased by about 130 g/day for the past 20 years from 64.50 g/day in 1998 to 123.39 g/day in 2008, and to 198.01 g/day in 2018. The intake from eating at “institutional places” increased from 0.38 g/day to 6.62 g/day and 7.47 g/day, but such an increase was not significant. In terms of the intake by year according to “food insecurity,” the “mildly food insecure’” group showed a significant increase from 271.78 g/day in 2008 to 333.44 g/day in 2018, but the “enough food secure” group showed no significant increase. However, the “moderately food intake” and “severely food intake” groups showed a tendency of decreasing fruit and vegetable intake by year even though it was not significant. In terms of eating-out frequency, although the “≥1/day” group showed no increase in intake, the “‘seldom (<1/month)” group, which had the lowest eating-out frequency, showed a significant increase from 234.79 g/day in 1998 to 253.02 g/day in 2008, and 298.45 g/day in 2018. The “1–6 times a week” group showed a significant increase after adjusting *p* for trend. 

### 3.6. General Characteristics and Dietary Factors That Satisfy the Plant Food Intake Guidelines (≥400 g)

Table 6 shows the matters on survey subjects with General characteristics and dietary factors that satisfy the plant food intake guidelines (≥400 g). Overall, for all variables, the ratio of subjects significantly increased by 0.1–18.0%p (percentage points) over the past 20 years from 1998 to 2018, except some variables including eating at commercial location, college, or higher level of education, “severely food insecure” of food insecurity and eating-out frequency (*p* < 0.05). In detail, the ratio of consuming more than 400 g/day of “fruits and vegetables” showed a significant increase from 21.78% in 1998 to 24.63% in 2008, and 33.06% in 2018. There was a significant increase in both males and females, from 20.02% in 1998 to 37.65% in 2018 for males and from 22.88% in 1998 to 29.57% in 2018 for females. In terms of age, while the intake showed a significant increase in both age groups from 65 to 74 and 75 or older, the ratio of subjects consuming 400 g/day or more in the group aged 75 or older was relatively lower at 23.67% than that of the group aged 65–74 at 40.04%. In terms of residential area, the ratio of the subjects consuming 400 g/day or more was significantly increased both in urban areas and in suburban/rural areas. Moreover, in terms of employment status, a significant increase was observed in all groups (*p* < 0.001). In terms of education level, only subjects with the lowest level of education in the “middle school or lower” group showed a significant increase. The “high school” group showed an insignificant increasing trend. The “college or higher” group did not show any increasing trend. In terms of weight, the “underweight” and “obesity” groups showed no significant increase, but the “normal” and “overweight” groups showed a significant increase in the ratio of subjects consuming 400 g/day or more each day. Although there was no significant difference from 2008 to 2018 according to chewing ability, the better the chewing ability, the higher the ratio of subjects consuming 400 g/day or more. In terms of household income, only the “high” group showed no significant difference while the “low,” “middle-low,” and “middle-high” groups demonstrated a significant trend by year. All groups of “single-person households” and “multi-person households” showed an increase in the ratio of the subjects consuming 400 g/day or more. Looking the pattern of daily meals, the “B+L+D” group having three meals a day showed a significant increase in the ratio of subjects consuming 400 g or more over the 20 years from 26.29% in 1998 to 27.98% in 2008, and 40.14% in 2018. There was no significant increase observed in other groups. As for snacks, there was no significant increase in the intake ratio by year. Looking at the pattern of cooking location, the “C + I (commercial place + institution)” group showed a significant increase in the ratio of subjects consuming 400 g/day or more from 28.88% in 2008 to 69.51% in 2018. In terms of food insecurity, the “mildly food insecure” group showed a significant increase from 21.99% in 2008 to 29.09% in 2018, but other groups showed no significant difference. In terms of eating-out frequency, the “1–6 times a week” group showed an increase from 31.12% in 1998 to 30.07% in 2008, and 39.83% in 2018, the “seldom (<1/month)” group also showed a significant increase in the ratio. 

### 3.7. Factors Related to Fruit and Vegetable Intake (≥400 g/day) in the Korean Elderly according to Survey Year Based on WCRF/WHO Guidelines

Table 7 shows the factors related to the fruit and vegetable intake of the elderly Koreans by year based on the WCRF/WHO guidelines. In 1998, an intake of 400 g/day or more fruits and vegetables in female subjects was approximately 2.51 times (OR = 2.509) more than that in male subjects. In terms of education level, the intake in the college or higher group was about 3.83 times (OR = 3.831) that in the middle school or lower group. As for the daily meal pattern, intake in the group eating only breakfast and dinner decreased by 62.9% (OR = 0.371) compared to that in the group eating breakfast, lunch and dinner, and the intake in the group eating only breakfast and lunch decreased by 70.0% (OR = 0.300). Subjects consuming 400 g/day or more fruits and vegetables in the group eating snacks were about 6.51 times (OR = 6.510) as many as those in the group not eating snacks. In 2008, 10 years later, the ratio of consuming 400 g or more fruits and vegetables decreased by 37.3% (OR = 0.627) in the group aged 75 or older compared to that in the group aged 65–74. In terms of weight, it increased by about 1.74 times (OR = 1.735) in the obesity group compared to that of the normal weight group. As for the daily meal pattern, it decreased by approximately 64.7% (OR = 0.353) in the group eating only lunch and dinner compared to that in the group eating breakfast, lunch, and dinner, and it decreased by about 53.6% (OR = 0.464) in others. In addition, the number of subjects eating more than 400 g of fruits and vegetables was found to be higher by about 4.17 times (OR = 4.169) in the group eating snacks than in the group not eating snacks. As for the pattern of cooking location, it increased by about 2.38 times (OR = 2.381) in the group eating at home and commercial locations than in the group eating only at home. In terms of food stability, the mildly and moderately food insecure groups showed a 40.8% (OR = 0.592) and a 52.8% (OR = 0.472) decrease compared to that of the enough food secure group.

In 2018, 20 years later, the ratio of 400 g/day or more fruit and vegetable intake decreased by about 41.0% (OR = 0.590) in the group aged 75 or older compared to that in the group aged 65–74 years. In terms of education level, it increased by about 2.78 times ((OR = 2.780) in the college or higher group compared to that in the middle school or lower group. As for the daily meal pattern, it decreased by about 71.6% (OR = 0.284) in the group eating only lunch and dinner compared to that in the group eating breakfast, lunch, and dinner; it decreased by about 43.5% (OR = 0.565) in the group eating only breakfast and dinner; and it decreased by about 72.7% (OR = 0.273) in other groups. In addition, the number of subjects eating more than 400 g/day of fruits and vegetables was found to be higher by approximately 6.29 times (OR = 6.290) in the group eating snacks than in the group not eating them. As for the pattern of cooking location, it increased by about 7.72 times (OR = 7.723) in the group with institutional food services or eating at commercial locations compared to that in the group only eating at home. In terms of food stability, the mildly food insecure group showed a 52.8% (OR = 0.472) decrease compared to the enough food secure group. In terms of weekly eating-out frequency, it increased by about 2.24 times (OR = 2.237) in the group eating out 1–6 times a week compared to that in the group eating out at least once a day.

## 4. Discussion

This study showed trends in the consumption of total vegetables, unsalted vegetables, salted vegetables, total fruits, fresh fruits, and candied fruits among the Korean elderly population and compared and analyzed the consumption of fruits and vegetables, general characteristics, and diet behavior of 3772 Korean elderly people aged over 65 years who participated in the KNHNAES in 1998, 2008, and 2018. Previous studies have suggested that vegetable and fruit intake can prevent chronic diseases, such as cancer, diabetes, and cardiovascular diseases because they contain essential vitamins, minerals, fiber, and biological compounds [31,32,33].

Therefore, sufficient intake of vegetables and fruit is very important to maintain good health. In addition, the Korean Comprehensive National Health Promotion Plan [10] recommends the intake of 500 g/day or more vegetables and fruits per day as one of the measures to induce proper nutrition management and balanced diet and to promote the prevention and proper management of chronic diseases.

The subjects consuming five or more servings of vegetables and fruits a day, as recommended by the United States Department of Agriculture (USDA) guidelines based on the National Health and Nutrition Examination Survey (NHANES) data, was reported to be about 23.6% [34]. However, a study conducted on the 5 servings of vegetables and fruits per day (80 g/serving) in all ages using KNHANES in 2008 reported that the rate of satisfactory intake increased with age: 13.5% in subjects aged 13–18, 19.1% in those aged 19–39, 24.0% aged 49–59, and 25.4% aged 60 or older, which was similar to our result of 24.6% in 2008 [16]. 

For subjects aged 65–74 years in this study, the average intake increased significantly each year to reach 407.7 g/day in 2018, exceeding the standard of 400 g/day or more as prescribed in the WRCF individual guidelines [9]. However, in case of this study, no significant increase was found in the group aged 75 years or older, and the ratio satisfying the guideline of 400 g/day or more was 23.67% with an average intake of 285.9 g/day, which was much lower in this group than in the group aged 65–74. It seems necessary to establish a dietary education program and guidelines to emphasize the importance of fruit and vegetable intake for people 75 or older.

While it is well known that increased intake of vegetables and fruits leads to good health, fruit and vegetable intake is particularly affected by social and economic factors. Previous studies reported that the intake of vegetables and fruits increases with household income, education level, and age, and it tends to be higher in females than in males [35]. Among elderly Canadians aged 65 or older, the ratio of people consuming fruits and vegetables 5 times a day was higher in females with a ratio of 51–53% than in 35–43% in males in all age groups [36], because more females perceived vegetables and fruits as healthy foods [37] and had more awareness and greater self-motivation than males [38]. Previous studies reported that females are more closely related to higher household incomes and education levels [39,40,41,42]. In the results of this study, the intake of 400 g/day or more fruits and vegetables was about 2.51 (OR = 2.509) times higher in females than in males in 1998. However, there was no difference between men and women in 2008 and 2018.

According to previous studies [43,44,45,46] looking at the relationship between education level and fruit and vegetable intake, higher education levels affected the vegetable and fruit intake with low energy density and diet diversity [47,48], which could be explained by education cultivating the sense of chronic disease prevention and healthy eating [40]. In our results, the higher the education level, the higher the intake of fruits and vegetables. These results suggest the needs for developing customized educational programs to increase the intake of fruits and vegetables.

Furthermore, a study on the vegetable and fruit intake among adults over 30 years and meeting the World Cancer Research Fund (WCRF) standard of 400 g/day or more reported that the vegetable and fruit intake was significantly affected by household income [9,47]. As a result of examining the trends in vegetable and fruit intake of 400 g/day or more by household income in this study, there was no difference in the odds ratio according to income level in all survey years, in contrast to the results of previous studies. Further in-depth research seems to be required to investigate whether this applies only to elderly subjects.

Based on these results, even in the elderly population, vegetable and fruit intake differs according to household income and level of education. A previous study [47] reported the difficulty of increasing vegetable and fruit intake in the low-income group due to the price burden, low storability of vegetables and fruits, and insufficient storage space. Moreover, following global trends, the number of elderly single-person households in Korea is increasing due to the extension of the average lifespan, and due to changes in the perception of marriage [17]. Single-person households have been gradually increasing from 15.87%p (percentage points) in 1998 to 13.41% in 2008, and to 20.24% in 2018. Regardless of the increase in family size, fruit and vegetable intake tended to increase in all subjects over the past 20 years. This shows a similar trend to the results of previous studies [34]. However, the WHO/WCRF guidelines for plant food intake were still less than half of the subjects. It is thought that a policy or publicity plan should be prepared for the elderly to increase their intake beyond the standard intake in the guidelines.

Vegetable and fruit intake according to daily meal pattern was significantly lower in the other groups than in the group eating breakfast, lunch, and dinner. While the fruit and vegetable intake significantly increased for “breakfast” and “lunch,” no significant difference was found in “dinner,” but there was a significant increase from 107.31 g/day in 1998 to 109.59 g/day in 2008, and to 159.06 g/day in 2018. It can be observed that fruit intake is not restricted to a specific time, as the ratios of fruit intake in the morning and lunch are increasing despite the conventional habit of eating fruit in the evening. Compared to the group not eating snacks, the proportion of subjects consuming 400 g/day or more fruits and vegetables in the group eating snacks was higher by about 6.51 times (OR = 6.510) in 1988, 4.17 times (OR = 4.169) in 2008, and 6.29 times (OR = 6.290) in 2018, indicating a high ratio of fruit and vegetable consumption as snacks. Looking at the results of this study related to the intake of vegetables and fruits according to the cooking location of daily meal, the ratio of vegetable and fruit intake decreased at home but increased at commercial places in general. In 2008, the intake of vegetables and fruits increased about 2.38 times (OR = 2.381) in the “H+C (home + commercial place)” group compared to that of the group eating only at home and in 2018, about 7.72 times (OR = 7.723) in the “C+I (Commercial place + Institution)” group. Such results suggested that vegetables and fruits were more likely to be consumed in commercial places than in institutions and at home, which indicates an increase in the number of elderly people who enjoy commercial dining. 

There are several limitations in this study, including that vegetable and fruit intake was analyzed using only the 24-h recall data. Because the 24-h recall data include only the record of foods consumed for 24 h the day before the investigation, it is difficult to determine the general daily food intake based on information sampled for only one day [17,49,50]. However, despite such limitations, the strength of this study is that it includes a large number of participants, targeting a nationally representative population. To conduct more in-depth future research, KNHANES needs to be conducted for three days, twice on weekdays, and once on weekends [49] as proposed in previous studies. Such developments of survey data will provide more accurate and complete dietary survey results not only for the elderly but also for Koreans as a whole.

## 5. Conclusions

Summarizing the results of this study, in the past 20 years, the intake of fruits and vegetables among elderly Koreans over the age of 65 has increased from 268.27 g in 1998 to 355.8 g in 2018, an increase of about 86.53 g over the past 20 years. In addition, plant food intake recommended by the World Health Organization (WHO) and the World Cancer Research Fund (WCRF) has increased from 21.78% in 1998 to 33.06% in 2018, an increase of approximately 11.28%p (percentage points) over the past 20 years. It is thought that the results of this study can be used as basic data for establishing dietary policy. Furthermore, it is thought that it can be used in nutrition education and dietary guidelines development to increase fruit and vegetable intake from a sustainable perspective.

## Figures and Tables

**Table 1:** Intake of Fruit and Vegetable according to survey year.

Intake, g/day	1998 *n* = 919		2008 *n* = 1355		2018 *n* = 1448		Unadjusted *p* for Trend ^(2)^	Adjusted *p* for Trend ^(2), (3)^
	Mean	SE ^(1)^	Mean	SE	Mean	SE		
Total food	1011.18	24.64	1029.45	23.25	1250.52	22.77	<0.001 (+)	<0.001 (+)
Total vegetable	276.09	9.74	296.72	8.31	299.43	6.68	0.0219 (+)	0.4317 (+)
Unsalted vegetable	133.02	5.79	162.95	6.79	181.66	5.83	<0.001 (+)	<0.001 (+)
Salted vegetable	143.07	7.41	133.76	4.71	117.76	3.33	0.0008 (−)	<0.001 (−)
Total Fruits	136.79	12.55	127.50	8.15	179.07	9.57	0.0005 (+)	0.0035 (+)
Fresh fruits	135.24	12.40	124.92	7.87	174.14	9.52	0.0010 (+)	0.0073 (+)
Candied fruits	1.55	0.74	2.58	0.81	4.93	0.72	0.0007 (+)	0.0007 (+)
Fruit and vegetable ^(4)^	268.27	14.20	287.87	10.10	355.80	12.55	<0.001 (+)	<0.001 (+)
**Ratio (%, SE)** ^(5)^								
Total vegetable	27.42	0.68	28.65	0.59	24.38	0.45	<0.001 (−)	<0.001 (−)
Unsalted vegetable	13.11	0.43	15.29	0.46	14.40	0.39	0.1041 (+)	0.0886 (+)
Salted vegetable	14.31	0.57	13.36	0.45	9.98	0.28	<0.001 (−)	<0.001 (−)
Total Fruits	11.00	0.80	10.20	0.60	13.00	0.52	0.0143 (+)	0.0109 (+)
Fresh fruits	10.87	0.80	9.98	0.59	12.59	0.52	0.0322 (+)	0.0263 (+)
Candied fruits	0.13	0.06	0.21	0.06	0.40	0.06	0.0006 (+)	0.0004 (+)
Fruit and vegetable	23.98	0.78	25.27	0.64	27.00	0.60	0.0014 (+)	0.0009 (+)

^(1)^ SE: Standard Error. ^(2)^ Unadjusted and adjusted *p* for trend were calculated by SURVEYREG procedure of SAS. ^(^^3)^ Adjusted for gender, age and energy intake. ^(^^4)^ Fruit and vegetable = fresh fruit intake + unsalted/non−starchy vegetable intake. ^(^^5)^ (Each food intake/Total food intake) × 100.

**Table 2:** General characteristics of subject sample by survey year.

	1998 *n* = 919		2008 *n* = 1355		2018 *n* = 1448		*p*-Value ^(2)^
	*n*	% ^(1)^	*n*	%	*n*	%	
**Gender**							
Male	363	38.21	536	41.39	597	43.29	0.0654
Female	556	61.79	819	58.61	851	56.71	
**Age**							
65~74y	646	67.10	920	66.12	830	57.40	<0.0001
75y+	273	32.90	435	33.88	618	42.60	
**Family size**							
Single-person	151	15.87	231	13.41	336	20.24	0.0003
Multi-person	768	84.13	1119	86.59	1112	79.76	
**Area**							
City	365	56.69	728	66.46	1069	78.66	0.0003
Rural area	554	43.31	627	33.54	379	21.34	
**Job**							
Inoccupation	591	69.76	837	69.94	890	67.87	0.6568
Occupation	328	30.24	471	30.06	447	32.13	
**Education level**							
Middle school or lower	830	88.79	1114	81.87	941	67.35	<0.0001
High school	64	8.36	127	10.66	245	20.46	
College or higher	25	2.85	71	7.47	151	12.19	
**Weight status**							
Underweight	94	10.71	71	5.45	27	1.71	<0.0001
Normal	376	43.76	470	35.62	464	33.79	
Overweight	173	20.26	330	26.66	361	27.09	
Obesity	206	25.27	425	32.27	523	37.41	
**Chewing ability**							
Very uncomfortable	-	-	399	30.48	145	9.21	<0.0001
Uncomfortable	-	-	400	29.43	405	28.49	
Normal	-	-	109	9.66	266	19.14	
Not uncomfortable	-	-	197	15.45	235	17.43	
Not uncomfortable at all	-	-	202	14.98	344	25.73	
**Household Income**							
Low	473	47.38	676	49.18	691	45.14	0.076
Middle-low	208	22.05	332	25.87	375	27.23	
Middle-high	138	15.97	133	11.41	226	16.54	
High	100	14.60	139	13.54	153	11.09	

^(1)^ Weighted %, ^(2)^ *p*-value by chi-square.

**Table 3:** Fruits & vegetable intake ^(1)^ according to survey year by general characteristics.

Intake, g/day	1998 *n* = 919		2008 *n* = 1355		2018 *n* = 1448		Unadjusted *p* for Trend ^(2)^	Adjusted *p* for Trend ^(2)^^(3)^
	Mean	SE	Mean	SE	Mean	SE		
**Gender**								
Male	273.42	19.29	316.21	15.81	378.28	14.47	<0.001 (+)	0.0004 (+)
Female	265.08	13.99	267.86	10.90	338.65	17.35	<0.001 (+)	<0.001 (+)
**Age**								
65~74y	277.34	16.02	318.46	12.89	407.72	17.28	<0.001 (+)	<0.001 (+)
75y+	249.76	19.39	228.16	15.90	285.87	12.34	0.0234 (+)	0.0518 (+)
**Area**								
City	301.58	21.11	316.10	13.35	368.76	13.15	0.0007 (+)	0.0058 (+)
Rural area	224.65	15.81	231.91	12.63	308.08	27.88	0.0048 (+)	0.0014 (+)
**Job**								
Inoccupation	277.19	24.61	288.22	16.20	386.72	24.59	<0.001 (+)	<0.001 (+)
Occupation	264.40	14.44	287.09	13.89	352.31	15.26	<0.001 (+)	0.0002 (+)
**Education level**								
Middle school or lower	256.83	13.64	266.60	9.62	313.40	13.14	0.0007 (+)	0.0009 (+)
High school	293.04	39.90	331.88	28.52	420.33	24.96	0.0021 (+)	0.0012 (+)
College or higher	551.81	92.48	467.66	43.72	543.78	45.10	0.4401 (+)	0.7059 (−)
**Weight status**								
Underweight	205.92	23.88	217.13	42.22	294.91	45.95	0.1364 (+)	0.2194 (+)
Normal	265.32	20.34	252.07	12.96	376.81	24.94	<0.001 (+)	<0.001 (+)
Overweight	259.00	21.14	317.31	22.76	351.73	16.68	0.0034 (+)	0.0074 (+)
Obesity	307.80	24.72	317.09	17.51	357.95	18.92	0.0463 (+)	0.0305 (+)
**Chewing ability**								
Very uncomfortable	- ^(4)^	-	240.69	17.78	278.59	26.08	0.2472 (+)	0.3538 (+)
Uncomfortable	-	-	291.27	15.52	322.40	21.60	0.1953 (+)	0.2386 (+)
Normal	-	-	296.43	28.80	371.70	29.57	0.0425 (+)	0.2325 (+)
Not uncomfortable	-	-	331.02	26.39	421.97	33.27	0.0165 (+)	0.0054 (+)
Not uncomfortable at all	-	-	340.88	27.00	381.01	17.31	0.2162 (+)	0.2412 (+)
**Household Income**								
Low	236.53	15.05	260.37	13.40	308.02	17.65	0.0010 (+)	0.0001 (+)
Middle-low	247.69	25.09	297.24	20.97	370.03	18.49	0.0001 (+)	0.0001 (+)
Middle-high	316.78	30.53	276.78	25.18	412.78	35.77	0.0012 (+)	0.1355 (+)
High	349.25	49.51	403.55	24.65	398.26	28.77	<0.001 (+)	0.9950 (+)
**Family size**								
Single-person	231.03	19.91	234.34	17.52	333.20	25.94	<0.001 (+)	<0.001 (+)
Multi-person	275.29	16.53	295.79	11.31	361.54	13.08	<0.001 (+)	<0.001 (+)

^(1)^ Fruits & vegetable intake = fresh fruit intake+unsalted/non-starchy vegetable intake. ^(2)^ Unadjusted and adjusted *p* for trend were calculated by SURVEYREG procedure of SAS. ^(3)^ Gender was adjusted for age and energy intake; Age was adjusted for gender and energy intake; and other variables were adjusted for gender, age and energy intake. ^(3)^ In 1998, KNHANES didn’t investigate questions related to this variable.

**Table 4:** Dietary behavior of subject sample by survey year.

	1998 *n* = 919		2008 *n* = 1355		2018 *n* = 1448		*p*-Value ^(2)^
	*n*	% ^(^^1)^	*n*	%	*n*	%	
**Daily meal**							
**Breakfast**							
Skipped	88	9.99	188	14.21	218	15.95	0.0058
Eaten	831	90.01	1167	85.79	1230	84.05	
**Lunch**							
Skipped	188	21.31	216	16.93	277	19.37	<0.0001
Eaten	731	78.69	1139	83.07	1171	80.63	
**Dinner**							
Eaten	148	15.78	217	16.53	204	14.13	0.1875
Skipped	771	84.22	1138	83.47	1244	85.87	
**Snack**							
Yes	359	40.58	531	39.54	865	60.28	0.3374
No	560	59.42	824	60.46	583	39.72	
**Cooking location of daily meal**							
**Home**							
Not eaten	12	1.35	70	5.57	104	7.36	<0.0001
Eaten	907	98.65	1285	94.43	1344	92.64	
**Commercial place**							
Not eaten	699	72.89	729	53.08	409	27.48	<0.0001
Eaten	220	27.11	626	46.92	1039	72.52	
**Institution**							
Not eaten	885	95.90	1276	94.55	1342	92.72	0.025
Eaten	34	4.10	79	5.45	106	7.28	
**Food insecurity**							
Enough food secure	- ^(3)^	-	426	33.48	708	49.59	<0.0001
Mildly food insecure	-	-	625	45.25	674	46.32	
Moderately food insecure	-	-	223	16.28	56	3.52	
Severely food insecure	-	-	81	4.99	8	0.56	
**Eating-out frequency**							
≥1/day	47	6.17	47	3.69	79	6.16	<0.0001
1~6 times a week	79	9.51	284	22.38	629	43.95	
1~3 times a month	155	17.28	429	31.24	439	29.50	
Seldom (<1/month)	637	67.04	595	42.70	301	20.39	

^(1)^ Weighted %, ^(2)^ *p*-value by chi-square. ^(3)^ In 1998, KNHANES didn’t investigate questions related to this variable.

**Table 5:** Fruit and vegetable intake according to survey year by dietary behavior.

Intake, g/day	1998 *n* = 919		2008 *n* = 1355		2018 *n* = 1448		Unadjusted *p* for Trend ^(3)^	Adjusted *p* for Trend ^(3),^ ^(4)^
	Mean	SE	Mean	SE	Mean	SE		
**Fruit and vegetable** ^(**1**)^	268.27	14.20	287.87	10.10	355.80	12.55	<0.001 (+)	<0.001 (+)
**Daily meal**								
Breakfast	58.23	4.05	59.77	3.43	72.60	3.90	0.0035 (+)	0.0172 (+)
Lunch	50.71	3.94	58.74	2.91	62.80	2.72	0.0196 (+)	0.0843 (+)
Dinner	52.01	4.21	59.77	4.16	61.35	2.69	0.1207 (+)	0.3963 (+)
Snack	107.31	10.49	109.59	6.88	159.06	8.66	<0.001 (+)	<0.001 (+)
**Ratio (%, SE)** ^(**2**)^								
Breakfast	27.61	1.08	25.70	0.81	22.72	0.69	<0.001 (−)	<0.001 (−)
Lunch	22.83	1.09	25.33	0.85	21.66	0.82	0.1035 (−)	0.1173 (−)
Dinner	24.00	1.02	24.89	0.91	22.14	0.74	0.0479 (−)	0.0357 (−)
Snack	25.57	1.67	24.07	1.20	33.48	1.08	<0.001 (+)	<0.001 (+)
**Cooking location of daily meal**								
Home	198.39	10.49	157.08	6.27	150.32	6.43	0.0003 (−)	<0.001 (−)
Commercial place	64.50	10.46	123.39	7.22	198.01	9.43	<0.001 (+)	<0.001 (+)
Institution	5.38	1.89	6.62	1.15	7.47	0.99	0.3575 (+)	0.4513 (+)
**Ratio (%, SE)** ^(**2**)^								
Home	82.67	1.63	66.70	1.47	51.06	1.22	<0.001 (−)	<0.001 (−)
Commercial place	15.59	1.58	30.17	1.34	46.10	1.29	<0.001 (+)	<0.001 (+)
Institution	1.73	0.45	3.03	0.45	2.83	0.39	0.1649 (+)	0.1833 (+)
**Food insecurity**								
Enough food secure	- ^(5)^	-	345.42	18.87	388.38	17.51	0.0854 (+)	0.2439 (+)
Mildly food insecure	-	-	271.78	15.83	333.44	17.06	0.0050 (+)	0.0078 (+)
Moderately food insecure	-	-	224.14	18.01	219.77	35.12	0.9118 (−)	0.7927 (+)
Severely food insecure	-	-	255.64	43.91	197.61	71.37	0.4968 (−)	0.47189 (−)
**Eating-out Frequency**								
≥1/day	357.01	70.38	345.80	44.15	351.55	40.72	0.9651 (+)	0.4716 (+)
1~6 times a week	356.32	47.71	340.57	24.01	400.22	17.69	0.0621 (+)	0.0342 (+)
1~3 times a month	318.01	32.08	290.92	17.67	330.16	21.55	0.3433 (+)	0.1594 (+)
Seldom (<1/month)	234.79	13.03	253.02	13.77	298.45	22.05	0.0067 (+)	0.0003 (+)

^(1)^ Fruit and vegetable = fresh fruit intake + unsalted vegetable intake, ^(2)^ (Each food intake/Fruit and vegetable) * 100. ^(3)^ Unadjusted and adjusted *p* for trend were calculated by SURVEYREG procedure of SAS. ^(4)^ Adjusted *p* for trend was adjusted for gender, age and energy intake. ^(5)^ In 1998, KNHANES didn’t investigate questions related to this variable.

**Table 6:** Frequency and ratio of survey subject with intake level satisfying individual guideline of WHO and WCRF ^(^^1)^^(^^2).^

	1998 *n* = 919		2008 *n* = 1355		2018 *n* = 1448		*p*-Value ^(4)^
	*n*	% ^(3^^)^	*n*	%	*n*	%	
**Total**	185	21.78	324	24.63	484	33.06	<0.0001
**Gender**							
Male	70	20.02	146	27.79	221	37.65	<0.0001
Female	115	22.88	178	22.40	263	29.57	0.0065
**Age**							
65~74y	138	23.25	256	29.41	330	40.04	<0.0001
75y+	47	18.79	68	15.31	154	23.67	0.0106
**Area**							
City	94	25.95	208	28.02	371	33.96	0.0225
Rural area	91	16.34	116	17.91	113	29.75	0.0035
**Job**							
Inoccupation	117	21.82	199	24.44	299	32.97	0.0004
Occupation	68	21.70	115	25.26	158	36.09	0.0011
**Education level**							
Middle school or lower	155	19.83	241	22.00	266	27.52	0.0055
High school	15	29.85	43	32.22	104	40.78	0.2425
College or higher	15	58.94	33	45.63	87	58.19	0.2394
**Weight status**							
Underweight	12	13.20	10	15.36	7	30.98	0.144
Normal	73	20.91	98	20.38	153	34.97	<0.0001
Overweight	35	19.96	82	25.24	131	33.36	0.0196
Obesity	53	28.75	123	30.79	178	33.23	0.5777
**Chewing ability**							
Very uncomfortable	- ^(7)^	-	70	18.77	36	22.74	0.3825
Uncomfortable	-	-	99	23.98	125	28.47	0.2399
Normal	-	-	29	24.96	89	34.92	0.1277
Not uncomfortable	-	-	55	32.24	90	38.70	0.2838
Not uncomfortable at all	-	-	63	31.93	135	39.42	0.141
**Household income**							
Low	79	17.23	133	20.18	195	28.43	0.0005
Middle-low	37	17.41	83	25.09	138	34.69	0.0026
Middle-high	37	28.71	30	23.30	93	39.11	0.0212
High	32	35.57	60	43.81	56	36.99	0.4919
**Family size**							
Single-person	26	17.74	46	19.86	103	31.20	0.0035
Multi-person	159	22.55	275	25.27	381	33.54	0.0002
**Pattern of daily meal ^(5)^**							
B + L + D	142	26.29	244	27.98	365	40.14	<.0001
B + L	14	14.19	23	23.45	29	27.33	0.1699
B + D	19	17.00	23	19.10	42	24.46	0.4673
L + D	4	12.80	13	17.88	28	22.33	0.5273
Others	6	10.22	21	14.24	20	14.44	0.7657
**Snack**							
Yes	137	40.74	244	45.76	410	47.02	0.3059
No	48	8.84	80	10.82	74	11.88	0.4587
**Pattern of cooking location ^(6)^**						
Only Home	108	15.31	62	9.69	50	12.12	0.0902
Only Commercial location	- ^(8)^	-	13	20.97	15	20.41	0.8267
H + C	71	40.70	229	43.03	379	42.46	0.9204
H + I	3	13.38	5	12.71	5	13.49	0.9958
H + C + I	2	43.18	13	65.66	25	44.18	0.3998
C + I	-	-	2	28.88	10	69.51	<0.0001
**Food insecurity**							
Enough food secure	- ^(7)^	-	146	34.48	279	37.97	0.4023
Mildly food insecure	-	-	126	21.99	190	29.09	0.0331
Moderately food insecure	-	-	40	14.42	14	20.75	0.2708
Severely food insecure	-	-	12	15.88	1	10.01	0.6406
**Eating-out Frequency**							
≥1/day	10	29.80	16	33.68	24	26.52	0.7747
1~6 times a week	23	31.12	79	30.07	248	39.83	0.044
1~3 times a month	47	32.77	116	26.93	129	29.54	0.5803
Seldom (<1/month)	105	16.93	113	19.32	83	25.56	0.0376

^(^^1)^ WHO (World Health Organization) and WCRF (World Cancer Research Fund) recommended vegetable and fruit intake of 400 g/day or more. ^(^^2)^ In case of all survey subjects in this table, they were sufficient intake group of fresh fruit and non-starchy/unsalted vegetable intake (≥400 g/day). ^(3)^ Weighted %. ^(4)^ *p*-value was calculated by chi-square. ^(5)^ B: Breakfast, L: Lunch, D: Dinner. ^(6)^ H: Home, C: Commercial location, I: Institution. ^(7)^ In 1998, KNHANES didn’t investigate questions related to this variable. ^(8)^ No data.

**Table 7:** Factors related to fruit and vegetable intake (≥400 g/day) in Korean elderly by each year.

	1998	2008	2018
**Gender (Ref. = Male)**			
Female	2.509 (1.533–4.108) ^(1)(2) ^*	0.995 (0.679–1.459)	1.169 (0.795–1.718)
**Age (Ref. = 65~74y)**			
75y +	1.276 (0.753–2.160)	0.627 (0.397–0.989) *	0.590 (0.404–0.862) *
**Area (Ref. = Rural area)**			
City	0.889 (0.544–1.455)	0.710 (0.436–1.156)	1.190 (0.732–1.935)
**Job status (Ref. = Inoccupation)**			
Occupation	0.916 (0.555–1.513)	1.088 (0.687–1.724)	0.946 (0.617–1.452)
**Education level (Ref. = Middle school or lower)**			
High school	1.389 (0.578–3.337)	1.221 (0.651–2.288)	1.469 (0.901–2.395)
College or higher	3.831 (1.251–11.735) *	1.667 (0.826–3.364)	2.780 (1.446–5.344) *
**Weight status (Ref. = Normal)**			
Underweight	0.813 (0.391–1.688)	1.436 (0.669–3.081)	0.851 (0.173–4.184)
Overweight	0.932 (0.493–1.762)	1.008 (0.591–1.721)	0.883 (0.593–1.314)
Obesity	1.588 (0.876–2.880)	1.735 (1.051–2.863) *	1.009 (0.676–1.508)
**Household Income (Ref. = Low)**			
Middle-low	0.774 (0.380–1.576)	1.054 (0.697–1.596)	0.997 (0.663–1.499)
Middle-high	0.935 (0.495–1.767)	0.749 (0.369–1.522)	0.830 (0.515–1.338)
High	1.162 (0.528–2.555)	1.647 (0.863–3.143)	1.064 (0.531–2.131)
**Chewing ability (Ref. = Very uncomfortable)** ^(3)^			
Uncomfortable	- ^(6)^	1.041 (0.612–1.772)	0.864 (0.470–1.588)
Normal	-	0.710 (0.305–1.655)	1.074 (0.581–1.984)
Not uncomfortable	-	1.139 (0.599–2.163)	1.125 (0.605–2.093)
Not uncomfortable at all	-	1.134 (0.584–2.200)	1.301 (0.705–2.403)
**Family size (Ref. = Single person)**			
Multi-person	1.502 (0.778–2.903)	0.898 (0.514–1.571)	0.666 (0.419–1.057)
**Pattern of daily meal (Ref. = B + L + D)** ^(4)^			
L + D	0.304 (0.055–1.683)	0.353 (0.166–0.747) *	0.284 (0.133–0.605) *
B + D	0.371 (0.179–0.771) *	0.606 (0.267–1.374)	0.565 (0.330–0.966) *
B + L	0.300 (0.134–0.669) *	0.561 (0.268–1.176)	0.606 (0.315–1.166)
Others	0.321 (0.097–1.064)	0.464 (0.222–0.969) *	0.273 (0.132–0.565) *
**Snack (Ref. = No)**			
Yes	6.510 (4.213–10.058) *	4.169 (2.573–6.755) *	6.290 (3.766–10.505) *
**Pattern of cooking location (Ref. = Only home)** ^(5)^			
Only Commercial location	- (7)	1.566 (0.637-3.852)	1.032 (0.320-3.327)
C + I	-	1.267 (0.011-139.868)	7.723 (1.992–29.945) *
H + I	1.473 (0.692–3.138)	1.500 (0.462-4.868)	0.932 (0.20–4.184)
H + C	0.491 (0.092–2.608)	2.381 (1.346–4.210) *	1.430 (0.723–2.829)
H + C + I	2.316 (0.428–12.540)	3.897 (0.935–16.232)	1.010 (0.350–2.917)
**Food insecurity (Ref. = Enough food secure)** ^(3)^			
Mildly food insecure	- ^(6)^	0.592 (0.357–0.983) *	0.852 (0.605–1.199)
Moderately food insecure	-	0.472 (0.265–0.841) *	0.999 (0.402–2.484)
Severely food insecure	-	0.505 (0.196–1.302)	0.581 (0.097–3.498)

^(1)^ Odd ratio (95% Confidence Interval), ^(2)^ Adjusted for energy intake, ^(3)^ This variable was not investigated by KNHANES in 1998, ^(4)^ B: Breakfast, L: Lunch, D: Dinner, ^(5)^ H: Home, C: Commercial location, I: Institution. ^(6)^ In 1998, KNHANES didn’t investigate questions related to this variable. ^(7)^ No data, * *p* < 0.05.
